# Separation of Volatile Fatty Acids from Model Anaerobic Effluents Using Various Membrane Technologies

**DOI:** 10.3390/membranes10100252

**Published:** 2020-09-24

**Authors:** Áron Bóna, Péter Bakonyi, Ildikó Galambos, Katalin Bélafi-Bakó, Nándor Nemestóthy

**Affiliations:** 1 Research Institute on Bioengineering, Membrane Technology and Energetics, University of Pannonia, 8200 Veszprém, Hungary; bona.aron@sooswrc.hu (Á.B.); bakonyip@almos.uni-pannon.hu (P.B.); bako@almos.uni-pannon.hu (K.B.-B.); 2 Soós Ernő Research and Development Center, University of Pannonia, 8200 Nagykanizsa, Hungary; galambos.ildiko@sooswrc.hu

**Keywords:** volatile fatty acids, purification, reverse osmosis, nanofiltration, forward osmosis, supported liquid membrane, ionic liquid extraction

## Abstract

Effluents of anaerobic processes still contain valuable components, among which volatile fatty acids (VFAs) can be regarded and should be recovered and/or used further in applications such as microbial electrochemical technology to generate energy/energy carriers. To accomplish the separation of VFAs from waste liquors, various membrane-based solutions applying different transport mechanisms and traits are available, including pressure-driven nanofiltration (NF) and reverse osmosis (RO) which are capable to clarify, fractionate and concentrate salts and organics. Besides, emerging techniques using a membrane such as forward osmosis (FO) and supported liquid membrane (SILM) technology can be taken into consideration for VFA separation. In this work, we evaluate these four various downstream methods (NF, RO, FO and SILM) to determine the best one, comparatively, for enriching VFAs from pH-varied model solutions composed of acetic, butyric and propionic acids in different concentrations. The assessment of the separation experiments was supported by statistical examination to draw more solid conclusions. Accordingly, it turned out that all methods can separate VFAs from the model solution. The highest average retention was achieved by RO (84% at the applied transmembrane pressure of 6 bar), while NF provided the highest permeance (6.5 L/m^2^hbar) and a high selectivity between different VFAs.

## 1. Introduction

Volatile fatty acids (VFAs) are carboxylic acids with six or fewer carbon atoms that can be saturated or unsaturated, the most common forms being acetic, formic, propionic and butyric acid [1]. For industrial applications, VFAs are produced mostly via chemical routes [2], nevertheless, microbial fermentations offer an alternative [3]. VFAs are demanded by the chemical industry to synthetize alcohols, ketones, esters, olefins or aldehydes [4]. Furthermore, VFAs can be used in textiles and cosmetics or in biopolymer plastic applications. They can also be seen as a feedstock in biofuel production or microbial electrochemical technologies [5,6]. VFA separation/rejection can be carried out by reverse osmosis (RO) and nanofiltration (NF) (as pressure-driven) membrane processes. Both RO and NF are well-established, not just for the chemical and water treatment industry, but also for biotechnological downstreaming [7]. The low molecular mass and chemical properties of VFAs enable NF and RO to be plausible choices. RO membranes offer the tightest pores for pressure-driven processes and accordingly have the highest rejection for ionic species, along with a reasonable water flux and energy consumption thanks to the intensive R&D and optimization works of the past decades by the scientific and industrial community [8,9,10]. In RO, solution pH levels where VFAs are mainly in ionized forms seem to increase the retention properties up to 99.7% [11]. Still, Jänisch et al. found significant amounts of acetic acid in the permeate of various RO membranes fed by biogas plant hydrolysates [12].

NF membranes demonstrate several advantages compared to RO: the larger pore size and the typically higher surface charge are expected to enhance the selectivity (e.g., separation of divalent and monovalent salts), productivity and decrease the energy investment in the process. However, a trade-off is represented by lower rejections for most compounds [10,13,14]. Actually, Zacharov et al. measured the acetate and butyrate rejection of commercial NF membranes to be between 20–75%, which could be somewhat increased by adding salts to the system [15]. In fact, some process designs intentionally use the low VFA rejection of NF membranes to separate them from other organics, which can be sent back to the bioreactor and VFAs are recovered in the permeate [16,17]. Interestingly, Xiong et al. reported a superior, 100% rejection of butyric acid with NF membranes [18].

The Layer-by-Layer (LbL) method represents a unique NF membrane fabrication technique where polycations and polyanions are alternatingly layered on a charged substrate, typically an ultrafiltration membrane (UF) to build a dense amorphous polyelectrolyte multilayer (PEM), which acts as the active separation layer. The high charge density of the PEM leads to a high monovalent salt passage, a relatively greater rejection of organics and, if the membrane has a high surface charge, a high mono/divalent salt selectivity [19,20,21,22]. PEM membranes have further advantages compared to polyamide membranes, which originate from the different chemistry: a much higher resistance to oxidants, such as chlorine, a higher pH tolerance [23], high self-healing ability [24,25,26] and, if the substrate allows, solvent resistance [27,28]. The LbL method lends itself to produce NF membranes with a hollow fiber geometry: by using UF membranes with such geometry as substrates. Hollow fiber membranes have distinct advantages: a higher crossflow tolerance, no spacer fouling and the option of backwashing [29,30]. On the other hand, in the case of longer fibers, concentration polarization can cause significant issues [31], certain feed conditions can destabilize the top layer (very high salinity, surfactants) and the maximum recommended transmembrane pressure is only 6 bars (in the case of the products available on the market at the date of publication).

Forward osmosis (FO) is an osmotically driven separation process where the higher osmotic potential of the draw solution governs the water through a tight (comparable to RO) semipermeable membrane. To achieve an effective separation, not only does the feed side have to have a thin active layer, but the whole membrane has to be thin to mitigate dilutive internal concentration polarization [32,33]. In spite of some intrinsic advantages, the practical implementation of FO has not caught up with the vast number of scientific publications to date [34]. According to McGovern et al., it seems unlikely that FO will outperform RO for seawater desalination [35], however it might find uses for special separation applications, e.g., in the food industry [36,37] where fouling can pose serious limitations for pressure-driven membrane processes. Finding an economical and scalable draw solution is still the most important challenge for FO [38]. At the moment, RO is still the most commonly used method to recover the draw solution, which inherently leads to a somewhat more energetically demanding process than simple RO; nonetheless, severe fouling might justify this setup in some cases [39], as it was demonstrated by the treatment of various wastewater feeds [40]. For feed solutions having lower osmotic potential which need dewatering (e.g., juice concentration), seawater, especially concentrated seawater from desalination plants, provides an economic and environmentally friendly, low-carbon footprint solution with obvious geographical limitations. VFA solutions, including real wastewater, can be effectively dewatered by FO [41,42], thanks to the tight nature of the membranes.

Liquid–liquid extraction also represents a potentially efficient method for VFA separation; however, it requires the acidification of the feed, and furthermore, the regeneration of the extractant adds burden to the process. Additionally, the toxic effect on the fermentation culture is a possible issue in the case of many solvents [43]. Compared to traditional solvents, phosphonium-based ionic liquid (IL) extraction achieved a higher maximum VFA loading, leading to a higher efficiency [44,45,46,47], but 1-hexyl-3-methylimidazolium bromide was also successful in recovering lactic acid from wine [48].

Supported ionic liquid membranes (SILMs) can be formed by immobilizing ionic liquids in porous matrices [49,50,51,52,53]. Such membranes enable a continuous extraction and stripping process of VFAs via ionic liquids [53,54], which transport the extractants to the stripping solution on the other side of the membrane. The concentration-driven transport of the VFAs is facilitated through the ionic liquid membrane by circulating the feed on one side of the membrane, and a stripping solution (pure water in our case) on the other side. Contrary to the former three technologies above, where the VFAs are concentrated in the retentate, in SILM extraction the goal is to transport VFAs to the extractant side of the membrane from the feed side. The pure VFA solution obtained in this process can be concentrated by a second process step, e.g., by RO. Another major difference of SILMs—compared to pressure- and osmotically driven membranes—is that the passage on SILMs depends mainly on polarity (partition coefficients) and much less on molecular size [55]. This difference in selectivity based on size vs. polarity is clearly shown in the work of Abejón et al., where the selectivity between monosaccharides and macromolecules (lignosulfonates, lignin) are compared for SILM vs. loose NF and tight UF, displaying a difference of almost two orders of magnitude [56].

Membrane processes are able to recover VFAs by various methods [57], but the original research studies compare only one or two methods in the same scope. In this study, four separation processes (RO, NF, FO, SILM) are investigated by a comparative methodology supported by statistical analysis according to the anaerobic effluents treating needs. Microbial processes can produce different VFA solutions, which can vary by composition, concentration and pH. To study the effect of the varying feed solution, we have set up an appropriate experimental design to investigate the efficiency of each separation process.

## 2. Materials and Methods

### 2.1. Membranes and Chemicals

The RO membrane was a flat sheet, SW30 Polyamide, Thin Film Composite (TFC)(Dow Chemical, Midland, MI, USA). It was used after conditioning by ultrapure water filtration and left an additional day in wet conditions, allowing swelling. The dNF40 type nanofiltration membrane (NX Filtration BV, Enschede, Netherlands) in an MP025 size module, which is the tightest commercial PEM NF membrane to date. This hollow fiber polyelectrolyte multilayer membrane has an ID of 0.7 mm, an approximate molecular weight cutoff (MWCO) of 400 Da and a pure water permeance of 5–7 L/m^2^hbar. The module has 25 × 300 mm size, and 0.067 m^2^ membrane area. To support the IL, a Durapore porous hydrophobic polyvinylidene fluoride (PVDF) membrane was used (Millipore, Bedford, MA, USA). It has a pore size of 0.22 μm, porosity of 75%, average thickness of 150 μm after swelling in ionic liquid. According to our former study [58], this yields a membrane with an average 26.5 mg/cm^2^ ionic liquid content.

Certified analytical grade sodium hydroxide, acetic acid, propionic acid and butyric acid were obtained from Sigma Aldrich. Ultrapure water (conductivity below 1 µS/cm) was used in all experiments. Ninety-eight percent pure 1-Butyl-3-methylimidazolium hexafluorophosphate ionic liquid (BMIM-PF6) was purchased from IO-LI-Tech (IoLiTec-Ionic Liquids Technologies GmbH, Heilbronn, Germany). The feed solutions were synthetic aqueous solutions of volatile fatty acids, with the pH adjusted by NaOH.

### 2.2. Supported Ionic Liquid Membrane Preparation

To prepare SILMs, first a porous flat sheet supporting membrane (D = 6 cm) and 1 mL of the BMIM-PF6in a Petri dish were placed in a desiccator under vacuum for 1 h in order to remove the air and water from the membrane pores. This way the hygroscopic IL could penetrate without any change in its physicochemical properties. After 1 h, the membrane was transferred into the IL and was maintained under vacuum for another 24 h. Then the membrane was taken out of the desiccator and its surface was carefully dried with a soft tissue to remove excess IL.

### 2.3. Experimental Setups and Analytical Methods

The concentrations of VFAs were measured using a Waters 2690 RP-HPLC system equipped with a Waters 2996 PDA detector (Waters Corporation, Milford, MA, USA) at 210 nm wavelength.

**Figure 1:**
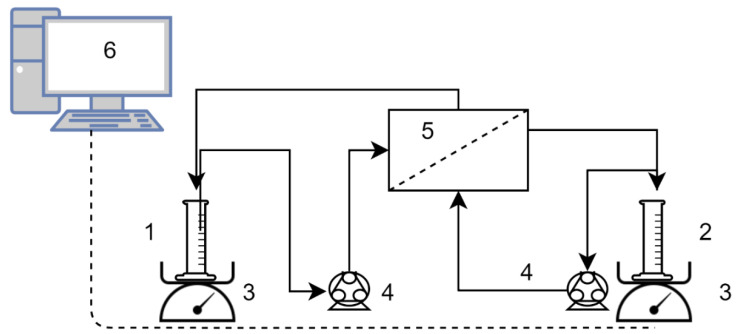
Setup of the forward osmosis (FO) tests. 1. Feed and 2. draw solution reservoirs, 3. digital scales, 4. peristaltic pumps, 5. membrane module, 6. data logging PC.

For the FO tests, the flat sheet SW30RO membrane was put in a custom-built membrane module with an active area of 150 cm^2^ in both side of the membrane with an active layer-facing feed solution. The setup was run with counter-current cross-flow and a peristaltic pump provided recirculation of the feed solution (FS) and draw solution (DS) in both sides of the membrane with a flow rate of 6 L/h. The DS and FS reservoir were placed on a digital balance connected to a computer to monitor and log the changes in the weight of the vessel as water permeates through the membrane to the DS side from the feed. A schematic diagram of the setup is presented in Figure 1.

NaCl solution with an average concentration of 1 M was used as the DS for tests, 50 mL of a 1.33 M NaCl solution was initially placed in the DS reservoir and tests were terminated by obtaining 100 mL permeate (dilution of a factor 3). Both solutions were tempered at 25 °C.

**Figure 2:**
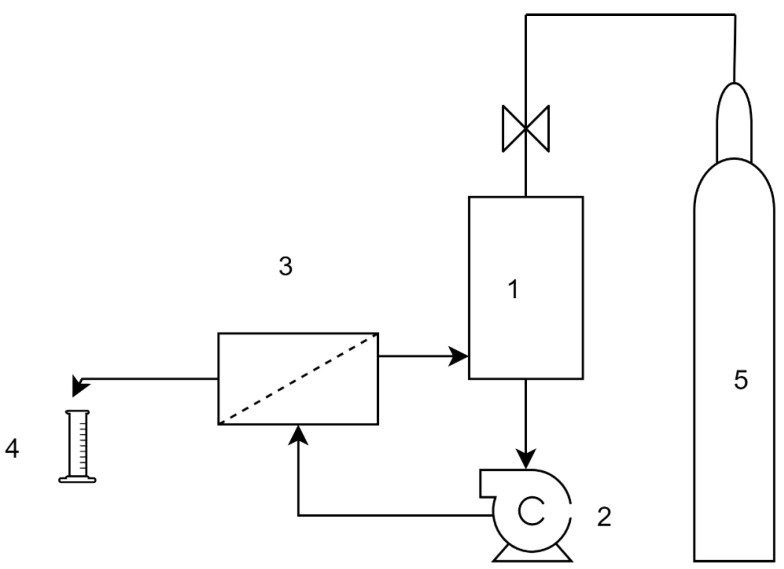
Setup of the reverse osmosis (RO) tests. 1. Pressurized feed tank, 2. centrifugal pump, 3. membrane module, 4. permeate collection, 5. nitrogen gas cylinder.

The RO test was carried out by a cross-flow, plate and frame membrane configuration system (3DTA) with a 0.015 m^2^ effective membrane area. [59]. The test system was used in batch mode 1 dm^3^ feed solution with 0.1 dm^3^ permeate collection (Figure 2). After, the measurement permeate samples were tested by the HPLC method. The transmembrane pressure was 6 bar, cross-flow velocity 1.0 m/s, and the feed temperature was kept at a constant 25 °C.

**Figure 3:**
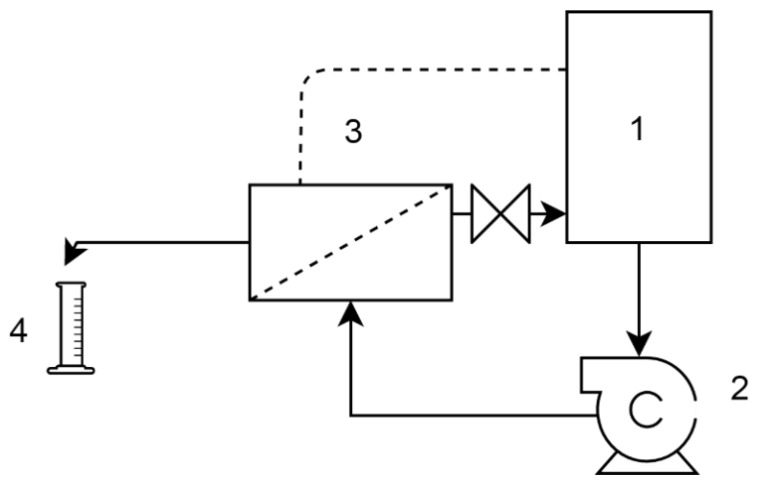
Setup of the nanofiltration (NF) tests. 1. Feed tank, 2. centrifugal pump, 3. membrane module, 4. permeate collection.

The NF membrane performance was evaluated using a cross-flow membrane filtration set-up (Figure 3). The transmembrane pressure was 5 bar, and the cross-flow velocity was 0.2 m/s. The system was equipped with a 1 L volume feed tank where the temperature was kept at a constant 25 °C by a tempered bath. The concentrate and permeate were recirculated into the feed tank for approximately 60 min and the stationary state was controlled by conductivity measurements before taking samples.

The SILM membranes containing IL were tested in a plate frame module with D = 5.5 cm; each compartment was 2.5 mm wide which gives 6 mL feed and receiver side volume. They were circulated with a peristaltic pump with 0.5 L/h flow rate from a 50 mL reservoir. Each measurement was carried out over 24 h at 25 °C. Water fluxes were lower than 1 mL during the 24 h tests.

### 2.4. Calculations

The permeance was calculated in liters per square meter per hour per bar (L/m^2^hbar) applying Equation (1):

(1) $$k=\frac{∆V}{Am*∆t*p}$$

where *k* is the permeance, Δ*V* is the total volume of the permeate (L) collected at Δ*t*, the filtration time (h), *Am* is the effective membrane filtration area (m^2^), and *p* is the applied transmembrane pressure.

The observed rejection was calculated using Equation (2), as follows:

(2) $$R_{i}\left(\%\right)=\frac{C_{f_{i}}-C_{p_{i}}}{C_{f_{i}}}*100$$

where *C_f_* and *C_p_* represent the solute content in the feed and permeate streams, respectively.

### 2.5. Experimental Design and Analysis

The aim of applying an experimental design was to identify which feed parameters (concentration of acetic, propionic and butyric acid and additionally the pH) of this separation process have a significant effect on VFA separation process output parameters, namely permeate flux and VFA rejections. In case of RO and NF permeance and retentions of VFA, for FO flux and retentions and for SILM membranes, the different VFA specific fluxes are the dependent variables. This method does not take into consideration the interaction between factors [60]. A first order, linear approach is sufficient for the screening procedure:

(3) $$V=\sum \;M_{i}X_{i}+I$$

where *V* is the response or dependent variable according to the membrane process, *M_i_* is the linear regression coefficient, *X_i_* is the level of the independent variable, and *I* is the model intercept. According to a 2-level, fractional-factorial experimental design (with 3 repetitions in the center point) all factors were screened at a low and a high level coded as (−1) and (+1), respectively (see Table 1). The design matrix is shown in Table 2 where the effect of 4 variables was investigated in 11 independent experimental runs including triplicate measurements in the center point to estimate the standard deviation. The sodium concentrations were determined by the VFA concentrations and the pH values. Statistica 8 software was used to analyze the experimental design. ANOVA and standardized effect (effect divided by standard errors) were calculated to determine statistical significance (with a *p*-value of 0.05). The measurements were carried out in random order.

## 3. Results

### 3.1. Reverse Osmosis

Results of the RO experiments are summarized in Table 3. The permeance varies around 2.5 L/m^2^hbar. The retention values of different VFAs are quite similar, acetic acid having the smallest average value (80%) and propionic and butyric acid having slightly higher values (84%).

Result of the statistical analysis can be found in Table 4. In case of permeance, no parameter has any significant effect. The results of individual acid retentions depend most strongly on pH, which has the strongest effect in case of propionic acid, making it significant. This is similar to what Masse et al. measured at higher pH values [61].

### 3.2. Nanofiltration

In the case of NF, the flux values are higher than RO, but divergences are also higher (see Table 5). According to this effect, the average retentions are lower (50%, 64%, 74%, acetic, propionic, butyric) but still interesting for industrial use, with a notable selectivity between the different compounds.

The effects (Table 6) are stronger than in the case of RO separation. All acid has a significant effect on flux, but none of them on the acid retention, which is strongly controlled by the pH value.

### 3.3. Forward Osmosis

In case of FO, permeances cannot be calculated because the salt concentration changes, therefore the flux data are presented at Table 7. The flux is low, hindered by internal concentration polarization, which lowers the rejection values for VFAs.

In the FO experiments, the permeance was affected by pH (Table 8). A higher pH resulted in a higher permeance as Jung et al. also found [41]. The retention of each acid was affected by the concentration of given compounds, although this effect was below statistical significance for acetic acid.

### 3.4. Supported Liquid Membrane

After preparing the SILM, the previous experimental design matrix measurement was performed. Both the receiver and the feed side concentrations were analyzed, and the individual acid fluxes were calculated (Table 9).

According to the statistical analysis (Table 10), this separation is led mostly by the concentration difference and there are no cross effects between the components. The pH has a slightly adverse effect in the case of propionic and butyric acid.

## 4. Discussion

All selected separation methods are capable of purifying VFAs from the model solution. The highest VFA rejection can be achieved by RO filtration, but this method has the highest energetic demand and intensive pretreatment requirements.

The rejection of VFAs is on the lower side in the case of NF compared to RO, especially considering the unusually low pressure applied for RO (with higher pressure, higher rejection can be expected). If the goal is also NaCl (or other monovalent salt) passage besides VFA retention, then further consideration of such LbL NF membranes might be worthwhile, especially if tighter LbL membranes become available commercially, such as the asymmetric membrane developed by te Brinke et al. [62].

The selectivities between the different VFA components of all four methods are compared by presenting the separation factors (α_ij_ = passage_i_/passage_j_) in Table 11. This represents only an approximate comparison between the membranes since the selectivity is also dependent on the feed concentrations. Passage on SILMs depends less the molecular size, and more on the polarity, so unsurprisingly the transport of butyrate is the most favored, which is not only the largest, but the most hydrophobic VFA in our study. The other three membranes exhibit an opposite selectivity, which is most likely governed by size.

The selectivity between the three VFAs was markedly larger in the case of LbL NF compared to RO, or to the acetate/butyrate selectivity measured by Zacharov et al. on TFC NF membranes [15]. This feature might be useful if the separation of the different acids is also a goal during the process.

Non pressure-driven systems, such as FO and SILM, are less prone to fouling and can treat direct wastewater streams as well. In this study, FO gives a lower selectivity than RO; however, the used membranes were same. Selectivity can be improved by providing appropriate flow conditions and choosing a membrane which exhibits lower internal concentration polarization. In the case of SILM, the goal is to maximize transport of VFAs to the extractant side from the feed side of the membrane, which is the opposite of the other three methods studied. It is also different regarding the absence of water flux. VFAs are purified but diluted which means another concentration step is needed. In our study, the typical VFA flux was around 1 g/m^2^h, which means the process speed is approximately two orders of magnitude slower compared to a concentration step with RO or NF, which means further optimization would be needed to make this process industrially relevant.

**Scheme 1:**
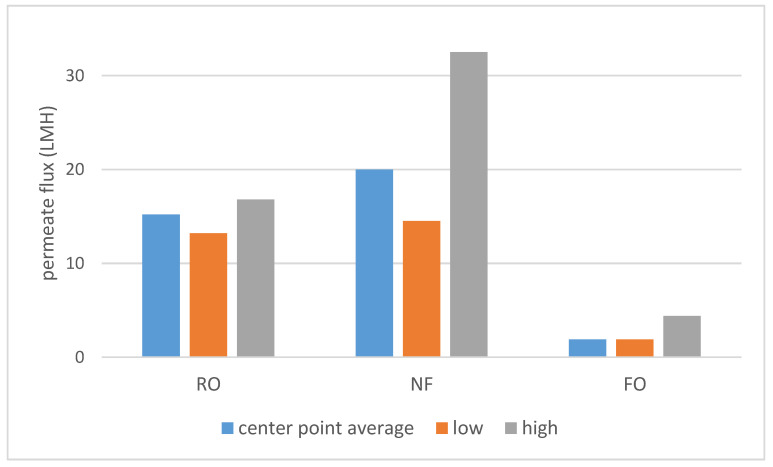
The center point (runs 9–11) average, lowest and highest measured permeate fluxes.

**Scheme 2:**
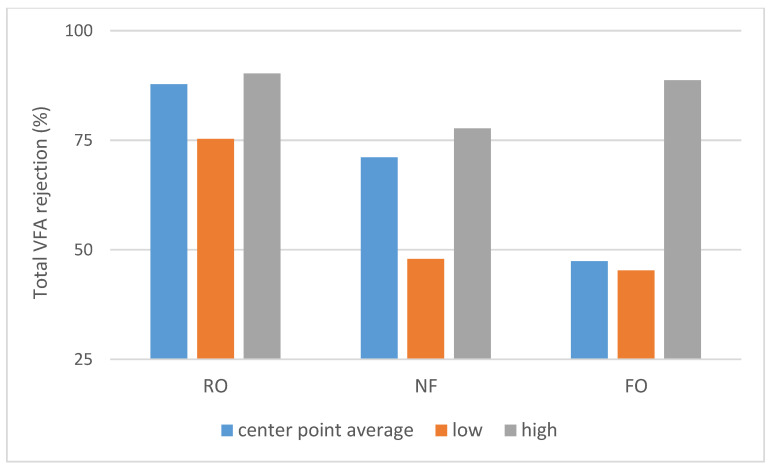
The middle point (runs 9–11) average, lowest and highest total VFA rejection values (R_total acid_).

As shown on Scheme 1, a large difference in permeate flux between FO and pressure-driven processes was observed. SILM was omitted from both Scheme 1 and Scheme 2 due to the different process properties. The dNF membrane exhibited a lower average rejection compared to RO (Scheme 2), which can be expected due to the looser membrane structure. On the other hand, the nominally 400 Da MWCO dNF40 membrane provided VFA rejection values, which are on the higher side for NF, the typical being 20–75% [15].

Summarizing the effect sizes of each system (see Table 12), RO and NF were mostly affected by pH, while FO and SILM were most affected by acid concentrations. This behaviour provides directions for future aplications: RO consistenly provides good rejection, with a small dependencene on pH. The rejection properties of NF membranes are more sensitive to feed conditions, particularly to pH, and furthermore a larger difference can be seen between the retention of different VFAs compared to RO, which opens the possibility to separate them via membrane technology.

Switching the RO membrane to an FO mode provided no significant advantage over the pressure-driven mode; however, this might be different in the case of real anaerobic wastewater because of different fouling mechanisms. SILM might prove to be a feasible solution if the feed pH is acidic and severe fouling takes place, and if the extraction speed can be significantly improved in the future.

Our study demonstrates that the dependence of membrane transport properties from the feed conditions indicates that pilot testing is essential for appropriate process design.

## Figures and Tables

**Table 1:** The codes and levels of the variables.

Variable	Low Level (−1)	High Level (+1)	Center Point (0)
Acetic acid (g/L)	0.3	1.5	0.9
Propionic acid (g/L)	0.1	0.5	0.3
Butyric acid (g/L)	0.6	3.0	1.8
pH	5.5	8.5	7.0

**Table 2:** The experimental design matrix, showing the feed concentrations and pH values.

Run	Acetic Acid (g/L)	Propionic Acid (g/L)	Butyric Acid (g/L)	pH	Na (g/L)
1	0.3	0.1	0.6	5.5	0.25
2	0.3	0.1	3.0	8.5	0.93
3	1.5	0.1	0.6	8.5	0.76
4	1.5	0.1	3.0	5.5	1.16
5	0.3	0.5	0.6	8.5	0.43
6	0.3	0.5	3.0	5.5	0.87
7	1.5	0.5	0.6	5.5	0.74
8	1.5	0.5	3.0	8.5	1.51
9	0.9	0.3	1.8	7.0	0.90
10	0.9	0.3	1.8	7.0	0.90
11	0.9	0.3	1.8	7.0	0.90

**Table 3:** RO permeance and retentions of acid according to experimental design.

Run	Permeance (L/m^2^hbar)	R_acetic acid_ (%)	R_propionic acid_ (%)	R_butyric acid_ (%)	R_total acid_ (%)
1	2.7	72.5	80.2	81.6	78.7
2	2.2	82.6	86.7	83.7	83.7
3	2.5	85.3	87.5	81.9	84.5
4	2.4	70.1	81.8	81.7	77.9
5	2.8	80.2	86.8	80.2	82.5
6	2.6	70.1	77.2	81.5	80.0
7	2.4	74.5	76.8	76.1	75.3
8	2.2	85.1	84.4	87.7	86.6
9	2.7	86.9	86.5	87.8	87.4
10	2.3	92.3	90.7	89.1	90.2
11	2.6	86.1	85.4	85.8	85.8

**Table 4:** Standard effect of parameters at RO experiments.

Variable	Permeance	R_acetic acid_	R_propionic acid_	R_butyric acid_	R_total acid_
Acetic acid	−0.507	0.479	−0.037	0.026	−0.064
Propionic acid	0.166	−0.034	−1.102	−0.295	−0.031
Butyric acid	−0.631	−0.231	−0.124	1.266	0.567
pH	−0.204	2.295	2.950 *	1.085	1.996

*: significant effect.

**Table 5:** NF flux and retentions of acid according to experimental design.

Run	Permeance (L/m^2^hbar)	R_acetic acid_ (%)	R_propionic acid_ (%)	R_butyric acid_ (%)	R_total acid_ (%)
1	6.5	48.7	52.7	65.1	59.0
2	4.1	47.0	70.5	81.0	77.7
3	4.7	55.9	71.0	84.1	64.3
4	3.5	43.0	47.0	55.1	51.0
5	4.8	62.9	75.5	83.5	76.2
6	3.7	46.4	52.4	61.5	59.1
7	4.1	42.2	52.1	58.7	47.9
8	2.9	43.6	67.4	80.2	67.9
9	4.0	53.5	68.8	81.9	72.1
10	3.9	49.8	68.2	80.8	70.3
11	4.1	50.2	70.0	81.4	70.9

**Table 6:** Standard effect of parameters at NF experiments.

Variable	Permeance	R_acetic acid_	R_propionic acid_	R_butyric acid_	R_total acid_
Acetic acid	−4.145 *	−1.632	−1.004	−0.719	−2.873 *
Propionic acid	−3.507 *	0.048	0.446	−0.080	−0.051
Butyric acid	−6.270 *	−2.381	−1.012	−0.758	0.584
pH	−1.382	2.338	5.817 *	4.866 *	4.856 *

*: significant effect.

**Table 7:** FO flux and retentions of acid according to experimental design.

Run	Flux (L/m^2^h)	R_acetic acid_ (%)	R_propionic acid_ (%)	R_butyric acid_ (%)	R_total acid_ (%)
1	2.2	42.7	46.0	69.7	52.8
2	4.4	36.7	22.0	78.3	45.7
3	3.1	60.7	26.0	62.3	49.7
4	1.9	32.3	24.0	80.7	45.7
5	2.6	26.7	87.6	60.7	58.3
6	2.3	64.7	91.2	96.1	84.0
7	2.2	95.7	83.6	86.7	88.7
8	2.5	77.2	76.8	86.9	80.3
9	1.9	43.1	17.3	75.4	45.3
10	1.9	44.5	18.7	76.2	46.5
11	1.9	53.1	25.3	72.8	50.4

**Table 8:** Standard effects of parameters at FO experiments.

Variable	Permeance	R_acetic acid_	R_propionic acid_	R_butyric acid_	R_total acid_
Acetic acid	−0.921	1.887	−0.570	0.775	0.684
Propionic acid	−1.071	1.824	3.465 *	2.579 *	3.416 *
Butyric acid	0.557	−0.296	−0.458	4.121 *	0.180
pH	2.163	−0.676	−0.507	−2.956 *	−1.080

*: significant effect.

**Table 9:** Supported liquid membrane (SILM) flux of acids according to experimental design.

Run	Flux_acetic acid_ (g/m^2^h)	Flux_propionic acid_ (g/m^2^h)	Flux_butyric acid_ (g/m^2^h)	Flux_total acid_ (g/m^2^h)	R_acetic acid_ (%)	R_propionic acid_ (%)	R_butyric acid_ (%)
1	0.105	0.036	0.257	0.399	13.4	13.7	16.3
2	0.084	0.036	1.154	1.274	10.7	13.7	14.7
3	0.398	0.034	0.213	0.644	10.1	13.0	13.5
4	0.439	0.035	1.306	1.779	11.2	13.4	16.6
5	0.080	0.191	0.237	0.508	10.2	14.6	15.1
6	0.087	0.160	1.178	1.425	11.1	12.2	15.0
7	0.420	0.196	0.232	0.848	10.7	15.0	14.8
8	0.500	0.158	1.290	1.948	12.7	12.1	16.4
9	0.229	0.110	0.772	1.111	9.7	14.0	16.4
10	0.259	0.117	0.659	1.035	11.0	14.9	14.0
11	0.211	0.104	0.761	1.077	8.9	13.2	16.1

**Table 10:** Standard effects of parameters in SILM experiments.

Variable	Flux_acetic acid_	Flux_propionic acid_	Flux_butyric acid_	Flux_total acid_
Acetic acid	14.061 *	0.003	1.303	7.975 *
Propionic acid	0.627	17.611 *	0.042	3.126 *
Butyric acid	1.075	−2.153	24.371 *	19.890 *
pH	0.099	−0.280	−0.484	−0.387

*: significant effect.

**Table 11:** The average separation factors of the four separation methods.

VFA (i)	RO		NF		FO		SILM	
	α_i/ac_	α_i/prop_	α_i/ac_	α_i/prop_	α_i/ac_	α_i/prop_	α_i/ac_	α_i/prop_
propionate	0.82	-	0.73	-	1.11	-	1.25	-
butyrate	0.85	1.04	0.51	0.71	0.49	0.44	1.41	1.13

**Table 12:** Statistical analysis of significant effects.

System	Permeance	R_acetic acid_	R_propionic acid_	R_butyric acid_	R_total acid_
RO	-	-	pH	-	-
NF	aa, pa, ba	-	pH	pH	aa, pH
FO	-	-	pa	pa, ba, pH	pa
SILM	n.a.	aa	pa	ba	aa, pa, ba

aa: acetic acid, pa: propionic acid, ba: butyric acid, n.a.: nonanalyzed.
